# Grape Leaf Disease Identification Using Improved Deep Convolutional Neural Networks

**DOI:** 10.3389/fpls.2020.01082

**Published:** 2020-07-15

**Authors:** Bin Liu, Zefeng Ding, Liangliang Tian, Dongjian He, Shuqin Li, Hongyan Wang

**Affiliations:** ^1^ College of Information Engineering, Northwest A&F University, Yangling, China; ^2^ Key Laboratory of Agricultural Internet of Things, Ministry of Agriculture and Rural Affairs, Northwest A&F University, Yangling, China; ^3^ Shaanxi Key Laboratory of Agricultural Information Perception and Intelligent Service, Northwest A&F University, Yangling, China; ^4^ College of Mechanical and Electronic Engineering, Northwest A&F University, Yangling, China; ^5^ Ningxia Smart Agricultural Industry Technology Collaborative Innovation Center, Yinchuan, China; ^6^ West Electronic Business, Co., Ltd., Yinchuan, China

**Keywords:** grape leaf diseases, convolutional neural networks, deep learning, image augmentation, disease identification

## Abstract

Anthracnose, brown spot, mites, black rot, downy mildew, and leaf blight are six common grape leaf pests and diseases, which cause severe economic losses to the grape industry. Timely diagnosis and accurate identification of grape leaf diseases are decisive for controlling the spread of disease and ensuring the healthy development of the grape industry. This paper proposes a novel recognition approach that is based on improved convolutional neural networks for the diagnoses of grape leaf diseases. First, based on 4,023 images collected in the field and 3,646 images collected from public data sets, a data set of 107,366 grape leaf images is generated *via* image enhancement techniques. Afterward, Inception structure is applied for strengthening the performance of multi-dimensional feature extraction. In addition, a dense connectivity strategy is introduced to encourage feature reuse and strengthen feature propagation. Ultimately, a novel CNN-based model, namely, DICNN, is built and trained from scratch. It realizes an overall accuracy of 97.22% under the hold-out test set. Compared to GoogLeNet and ResNet-34, the recognition accuracy increases by 2.97% and 2.55%, respectively. The experimental results demonstrate that the proposed model can efficiently recognize grape leaf diseases. Meanwhile, this study explores a new approach for the rapid and accurate diagnosis of plant diseases that establishes a theoretical foundation for the application of deep learning in the field of agricultural information.

## Introduction

The grape industry is one of the major fruit industries in China, and the total output of grapes reached 13.083 million tons in 2017. However, diseases in grape leaves have hindered the development of the grape industry and caused significant economic losses. Hence, the identification and diagnosis of grape leaf diseases have received extensive attention from orchard workers and experts on disease and pest control.

The current approaches for disease detection are based mainly on visual recognition. However, it not only is visual recognition a time-consuming and laborious task, but the recognition accuracy does not satisfy the requirement (Dutot et al., 2013). The resulting erroneous diagnosis will lead to the abuse of pesticides, which will destroy the growth environment of the grapes and damage the quality of the fruit. Hence, various spectroscopy techniques have been widely applied in plant disease diagnosis and monitoring. However, the requirement of bulky sensors and precise instruments leads to low efficiency and high cost (Mahlein et al., 2013; Lin et al., 2014). With the development of computer vision technique, researchers have proposed some plant disease recognition algorithms based on machine learning methods (Waghmare et al., 2016; Ali et al., 2017; Hamuda et al., 2017; Akbarzadeh et al., 2018; Griffel et al., 2018; Sharif et al., 2018; Kaur et al., 2019; Khan et al., 2019; Kour and Arora, 2019; Liu et al., 2019; Wang et al., 2019; Zhu et al., 2019; Mohammadpoor et al., 2020). However, the classification features in these approaches are selected based on human experience, which limits the generalizability of the models and the accuracies of these models are still not satisfy the recognition requirement. In contrast, convolutional neural network (CNN) can effectively avoid complex image pre-processing and employ shared weights to reduce memory consumption. CNN is still considered to be one of the optimal algorithms for pattern recognition tasks. Thus, using CNNs to identify early plant diseases has become a research focus of agricultural informatization. In (Mohanty et al., 2016; Zhang and Wang, 2016; Lu J. et al., 2017; Lu Y. et al., 2017; Khan et al., 2018; Liu et al., 2018; Geetharamani and Pandian, 2019; Ji et al., 2019; Jiang et al., 2019; Liang et al., 2019; Oppenheim et al., 2019; Pu et al., 2019; Ramcharan et al., 2019; Wagh et al., 2019; Zhang et al., 2019a; Zhang et al., 2019b; ), CNNs are extensively studied and applied to the diagnosis of plant diseases. According to these studies, CNNs can learn advanced robust features of diseases directly from original images rather than selecting or extracting features manually, which outperform the traditional feature extraction approaches.

In this paper, an innovative recognition approach for grape leaf diseases based on CNNs is presented. This approach aims at overcoming two main challenges: First, CNN models require a large amount of data for training. However, each grape leaf disease appears in different time period, and the time for collecting disease images is limited. Thus, there are not sufficient diseased grape leaf images for the model’s training. Second, the task of fine-grained image classification for grape leaf diseases is challenging, and models that are trained *via* transfer learning have difficultly realizing satisfactory performance. Therefore, the design of the optimal CNN structure for recognition grape leaf diseases is a daunting task.

The innovation of the paper lies in the application of the improved CNN algorithm for grape leaf disease recognition and the main contributions and innovations of this paper are summarized as follows:

- A grape leaf disease data set is established and lays an essential foundation for the generalization of the model. First, to enhance the robustness of the model, images of diseased grape leaves with complex and uniform backgrounds are collected. In addition, to alleviate the overfitting phenomenon of the model, the original diseased grape leaf images are processed *via* data augmentation technology to generate enough training images. Moreover, the digital image processing technology is used to simulate the images of grape leaf diseases in various environments, thereby greatly improving the generalization performance of the model.
- An improved CNN model is proposed for diagnosing grape leaf diseases. By analyzing the features of grape leaf diseased images, a novel deep convolutional neural network model, namely, the dense Inception convolutional neural network (DICNN), is proposed. Deep separable convolution is first used by DICNN to build the first two convolutional layers to reduce the number of parameters and prevent the overfitting problem of the model. Then, Inception structure is used to enhance the extraction performance for multiscale disease spots. Finally, the dense connection strategy is applied to the four cascade Inception structures for alleviating the vanishing-gradient problem, encouraging feature propagation and reuse.

According to the experimental results, the accuracy of the DICNN model reaches 97.22%, which is better than other classic models. In addition, after data augmentation, using a data set of 107,366 diseased images of grape leaves, the accuracy increases by 14.42%, thereby exhibiting stronger robustness and better recognition performance.

The remainder of the paper is organized as follows: *Related Work* introduces and summarizes related work. In *Generating the Grape Leaf Disease Data Set*, based on the image acquisition of natural grape leaves, abundant grape leaf images are generated with image processing technology. *Identification Model for Grape Leaf Diseases* introduces the DICNN model. *Experimental Results and Discussion* presents the experiments for evaluating the performance of the model and analyses the results of experiments. The last section presents the conclusions of the paper.

## Related Work

To reduce the damage of diseases, many researchers have made tremendous efforts to identify plant diseases. With the continuous development of machine learning algorithms, they have been widely utilized to identify plant pests and diseases.

In (Hamuda et al., 2017), Hamuda et al. proposed an automatic crop detection algorithm. The algorithm was used to detect cauliflowers from video streams in natural light under different weather conditions, and the detection results were compared with ground-truth data that were obtained *via* manual annotation. This algorithm realized a sensitivity of 98.91% and a precision of 99.04%. In (Akbarzadeh et al., 2018), Akbarzadeh et al. proposed an approach for classifying plants that was based on support vector machine. The data set was composed of spectral reflectance characteristics of corn and silver beets at 635, 685, and 785 nm, with a rate of 7.2 km/h. The experimental results demonstrated that the proposed algorithm effectively classified the plants with an accuracy of 97%. In (Wang et al., 2019), Zhang et al. proposed a cucumber powdery mildew recognition approach that was based on visual spectra. Through the classification and recognition of spectral features, the 450- to 780-nm visible light band was selected as the research range. Then, the SVM algorithm was utilized to build the classification model, and the radial basis kernel function was applied to optimize the model. The experiments results demonstrated that this model realized accuracies of 100% and 96.25% for cucumber healthy leaves and powdery mildew leaves, respectively, and the total accuracy was 98.13%. In (Waghmare et al., 2016), Waghmare et al. proposed a technique for identification of grape disease through the leaf texture analysis and pattern recognition. The system took a single leaf of a plant as an input and segmentation was performed after background removal. The segmented leaf image was then analyzed through high pass filter to detect the diseased part of the leaf. Finally, the extracted texture pattern was fed to a multiclass SVM. In (Mohammadpoor et al., 2020), Mohammadpoor et al. proposed an intelligent technique for grape fanleaf virus detection. Based on Fuzzy C-mean algorithm, the area of diseased parts of each leaf was highlighted, and then it was classified using SVM. In addition, K-fold cross validation method with k = 3 and k = 5 was applied to increase the diagnostic reliability of the system. Experimental results showed that the average accuracy of the system was around 98.6%. However, machine learning algorithms require cumbersome image preprocessing and feature extraction (Kulin et al., 2017; Zhang et al., 2018). In contrast, CNN can automatically distinguish and extract the discriminative features for image identification.

In recent years, CNNs have made major breakthroughs in computer vision. Therefore, using CNN to identify plant diseases has become a research hotspot in agricultural information technology. In (Khan et al., 2018), Khan et al. isolated the regions of infection from the background and utilized VGG and AlexNet to extract the features of infection regions. Experiments were conducted on a Plant Village and CASC-IFW, and a classification accuracy of 98.60% was realized. The experimental results demonstrated that the proposed model outperformed the available approaches with high-precision and high-recognition accuracy. In (Zhang et al., 2019), Zhang et al. proposed a cucumber disease identification algorithm that was based on AlexNet, namely, GPDCNN. The approach fused the contextual information effectively by combining global pooling layers *via* dilated convolution, which could optimize the convergence and increase the recognition rate. The GPDCNN model was trained on six common cucumber leaf diseases and a recognition accuracy of 94.65% was realized. In (Liang et al., 2019), Liang et al. proposed a rice blast diagnosis system that was based on CNNs. The model was trained on a data set of 5,808 diseased images, which included 2,906 positive samples, and realized satisfactory performances in terms of the recognition accuracy, AUC, and ROC. The experimental results demonstrated that the proposed model could extract more discriminative and effective high-level features than the traditional approaches of LBPH and Haar-WT. In (Zhang et al., 2019), Zhang et al. trained a three-channel CNN model for the recognition of tomato and cucumber leaf diseases. The approach utilized the three channels of RGB separately to use the color information and realized the automatic extraction of diseased features through color information. On the data set of tomato and cucumber leaf diseases, the proposed model outperformed the traditional approaches in terms of the classification accuracy. In (Wagh et al., 2019), Wagh et al. proposed an automatic identification system of grape diseases for the recognition of five diseases including powdery mildew, downy mildew, rust, bacterial spots, and anthracnose. Feature extraction and model training of the leaf images were performed using pre-defined AlexNet architecture. And experimental results showed that the model was able to accurately classify grape diseases. In (Ji et al., 2019), Ji et al. proposed a united convolutional neural networks architecture based on an integrated method. The proposed CNNs architecture, namely, UnitedModel was designed to classify common grape leaf diseases. UnitedModel was able to extract complementary discriminative features owing to the combination of multiple CNNs. And the experimental results had shown that UnitedModel realized the best performance on various evaluation metrics and achieved an average test accuracy of 98.57%.

According to these studies, CNNs have obtained satisfactory results in plant disease recognition. However, CNNs is rarely used in the field of grape leaf disease identification. In addition, most application-oriented image identification algorithms are based on popular transfer learning techniques, and few improvements have been made to the algorithms. Hence, an image identification model that is based on CNNs for grape leaf diseases is proposed in this paper.

## Generating the Grape Leaf Disease Data Set

### Data Acquisition

Since no suitable data set is available for the identification of grape leaf diseases, a large amount of time is dedicated to collecting images of diseased grape leaves. A total of 7,669 images of grape leaves are collected with a digital camera and belong to seven categories: anthracnose, brown spot, mites, black rot, downy mildew, leaf blight, and healthy leaves. The classes of anthracnose, mites, downy mildew, and healthy leaves are collected in fine weather from the grape planting experiment station of Northwest A&F University, Shaanxi Province, China. And this part of the data set includes a total of 4,023 images. The class of brown spot, black rot, and leaf blight are collected from publicly available data sets, and this part of the data set includes a total of 3,646 images. **Table 1** illustrates the detail of original grape leaf disease data set.

**Table 1:** Original grape leaf disease data set.

	Anthracnose	Brown spot	Mites	Black rot	Downy mildew	Leaf blight	Healthy leaves
Number of images	1,124	1,383	1,106	1,187	910	1,076	883

**Figure 1:**
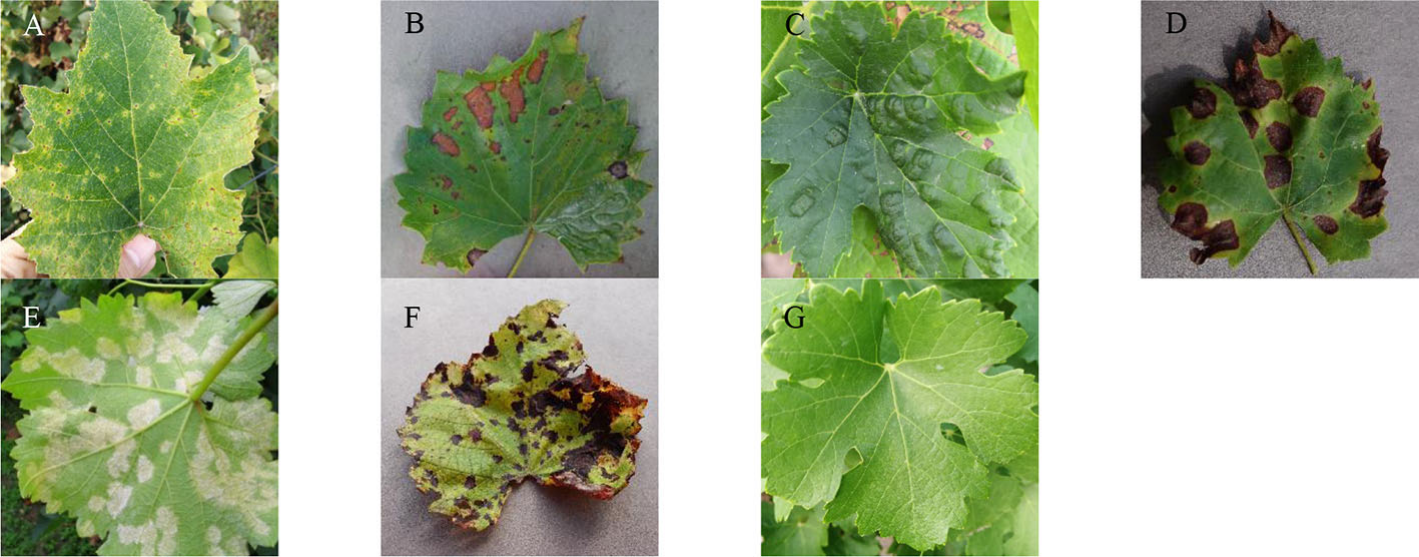
Seven common types of grape leaf images. **(A)** Anthracnose, **(B)** Brown spot, **(C)** Mites, **(D)** Black rot, **(E)** Downy mildew, **(F)** Leaf blight, **(G)** Healthy leaves.

Seven representative images of the data set are shown in **Figure 1**, where the differences among the seven types of images are clearly observed. The surface of a healthy grape leaf is green and has no spots. An anthracnose spot is nearly round. The central part of the spot is white, and the edge is dark purple. For the brown spot category, irregular brown spots are present on the surface of the grape leaves. The middle of each spot is dark brown, and the edges are brown. Mites cause many irregular white patches on the backs of the leaves, and the surface of the leaves are blistered. The spots of black rot are nearly round with a dark brown middle and brown edges. The yellow-green disease spots gradually appear on the fronts of the grape leaves with downy mildew, and white frosty mildew appears on the backs of the leaves. Leaf blight produces dark brown patches on the surface of grape leaves. The differences among these disease spots contribute to the recognition of various grape leaf diseases.

### Data Augmentation

The overfitting problem in the training stage of CNNs can be overcome *via* data augmentation. When random noise rather than the underlying relationship is fitted, the overfitting problem of deep learning models occurs (Heisel et al., 2017). With more images after expansion *via* data augmentation techniques, the model can learn as many irrelevant patterns as possible during the training process, thereby avoiding overfitting and enhancing the anti-interference ability under complex conditions.

Several digital image processing technologies are used to implement data augmentation operations. The effects of weather factors during shooting are simulated *via* image intensity interference, which include interference of brightness, contrast, and sharpness. Gaussian blur simulates the effects of hazy weather on image acquisition. The relative positions of the camera, and the diseased leaves are imitated *via* rotation transformations (including 90 degrees, 180 degrees, and 270 degrees) and *via* horizontal and vertical symmetry operations. Gaussian noise, interference of contrast, and sharpness are used to simulate the effects of equipment factors. In addition, PCA jittering is applied to expand the original data set.

The brightness values of each image are adjusted by randomly increasing or decreasing the RGB values of the pixels. Assume that *V*_0_ is the original RGB value, *V* represents the adjusted value, and *d* is the brightness transformation factor. The transformation process of the RGB value is expressed as:

$$V=V_{0}+\left(1+d\right)$$

Based on the median value of the brightness, the contrast value of the image is adjusted by increasing the larger RGB values and decreasing the smaller RGB values. The transformation process of the RGB values is expressed as:

$$V=i+(V_{0}-i)\left(1+d\right)$$

The Laplacian template is applied to the image to adjust the value of the sharpness. Assume that an RGB image pixel is represented as$c(x,y)={\left[R(x,y),G(x,y),B(x,y)\right]}^{T}$. The formula is as follows:

$$\nabla^{2}\left[c\left(x,y\right)\right]=\left[\begin{array}{c} \nabla^{2}R\left(x,y\right)\\ \nabla^{2}G\left(x,y\right)\\ \nabla^{2}B\left(x,y\right) \end{array}\right]$$

The image is rotated by rotating each pixel by the same angle around the center. Assume that P(*x*,*y*) is an arbitrary point in the image and that its new coordinate after clockwise rotation by *θ*° is P_2_(x,h-y). The calculated coordinates of the two points are expressed as:

$$\{\begin{array}{c} x=r\cos\alpha \\ y=r\sin\alpha \end{array}$$

$$\{\begin{array}{c} X=r\cos\left(\alpha -\theta \right)=x\cos\theta +yr\sin\theta \\ Y=r\sin\left(\alpha -\theta \right)=-x\cos\theta +yr\cos\theta \end{array}$$

The vertical symmetry operation uses the horizontal median line of the image as an axis to perform a symmetrical transformation on all pixels. Assume that *h* represents the height and P(*x*,*y*) is an arbitrary point in the image. After vertical symmetry processing, the coordinates of the new point are P_2_(x,h–y). The horizontal symmetry operation is similar to the vertical symmetry operation.

**Figure 2:**
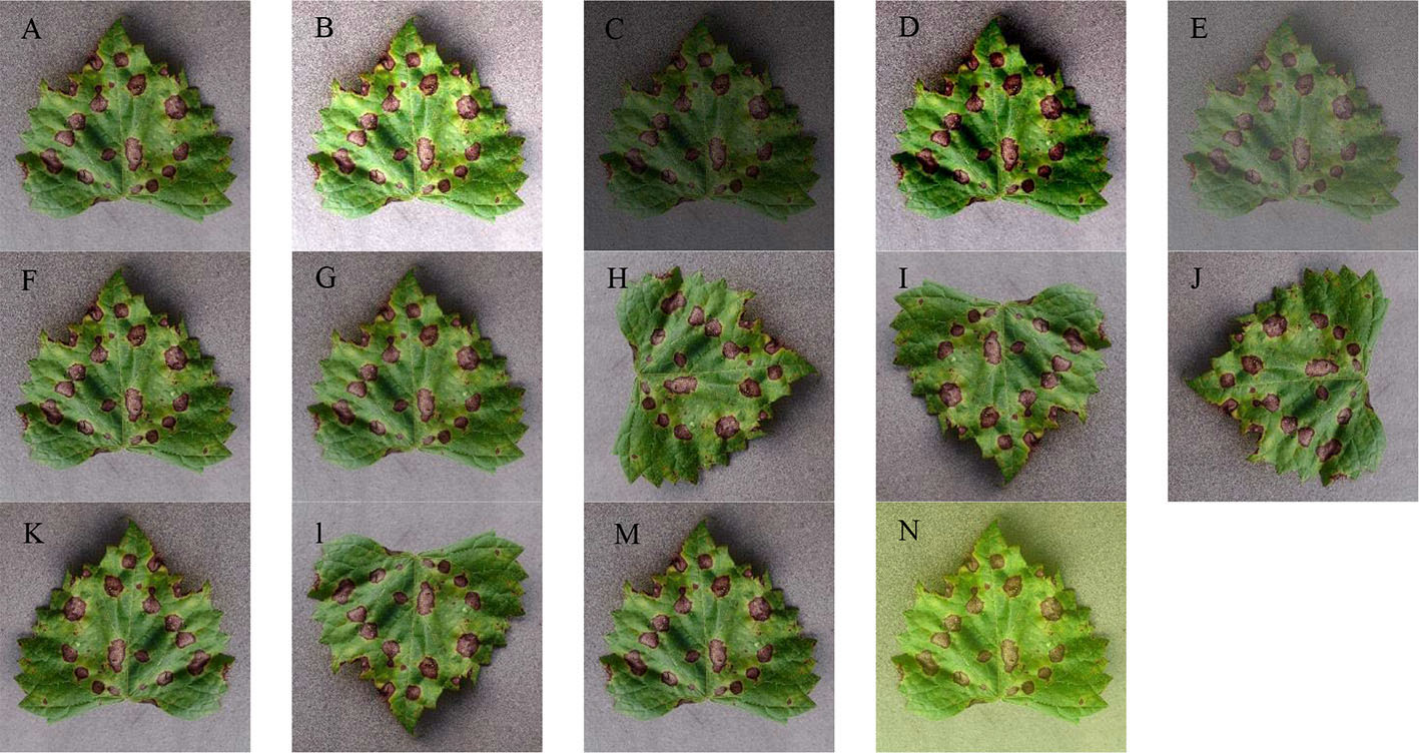
Image augmentation of a grape leaf disease image. **(A)** The original image, **(B)** high brightness, **(C)** low brightness; **(D)** high contrast; **(E)** low contrast; **(F)** high sharpness; **(G)** low sharpness; **(H)** 90 degree rotation; **(I)** 180 degree rotation; **(J)** 270 degree rotation; **(K)** vertical symmetry; **(L)** horizontal symmetry; **(M)** Gaussian noise, and **(N)** PCA Jittering.

Via these image generation techniques, 13 new images are derived from each image. **Figure 2** presents an example that illustrates the image generation process.

After the process of image augmentation, a data set of diseased grape leaf images has been obtained, and it includes 15,736 images from the anthracnose class, 19,362 images from the brown spot class, 15,484 images from the mite class, 16,618 images from the black rot class, 12,740 images from the downy mildew class, 15,064 images from the leaf blight class, and 12,362 images from the healthy leaf class. Then, all images in the data set are resized to 256 × 256. Finally, the data set is divided into three parts by the ratio of 6:2:2, which are, respectively, used as the training set, the validation set and the test set. Details on the data set are presented in **Table 2**.

**Table 2:** Grape leaf disease data set.

Class	Training images	Validation images	Testing images	Total number
Anthracnose	9,442	3,147	3,147	15,736
Brown spot	11,618	3,872	3,872	19,362
Mites	9,290	3,097	3,097	15,484
Black rot	9,970	3,324	3,324	16,618
Downy mildew	7,644	2,548	2,548	12,740
Leaf blight	9,038	3,013	3,013	15,064
Healthy leaves	7,418	2,472	2,472	12,362

## Identification Model for Grape Leaf Diseases

**Figure 3:**
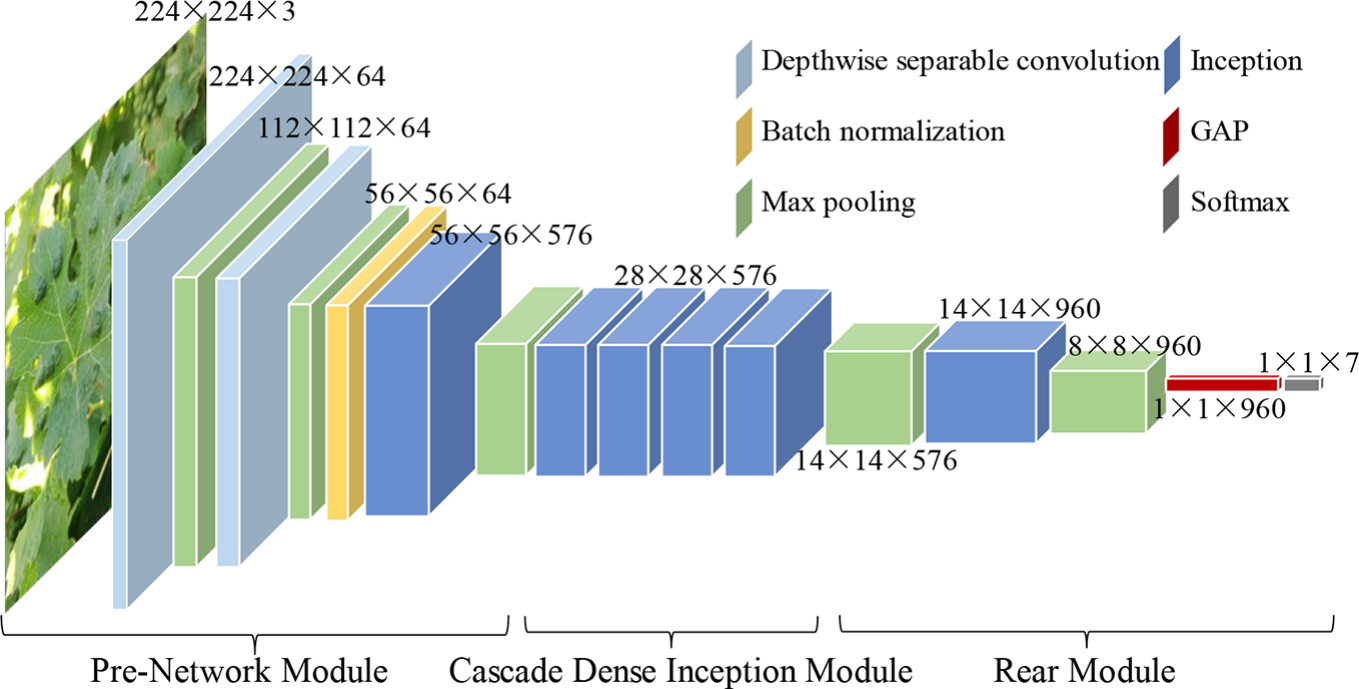
Structure diagram of the DICNN model.

Inspired by the architectures and performances of four classical CNN models, namely, VGG16 (Simonyan and Zisserman, 2014), GoogLeNet (Szegedy et al., 2015), ResNet (He et al., 2016), and DenseNet (Huang et al., 2017), a novel CNN-based model, namely, DICNN, is proposed for the diagnosis of seven common grape leaf classes. According to **Table 3** and **Figure 3**, the DICNN model includes three parts: the first part is the “pre-network module”, and its first deep separable convolutional layer is filtered with 64 kernels of size 3 × 3. Then, a 3 × 3 max-pooling layer is added after the first deep separable convolutional layer. The next deep separable convolutional layer contains 64 convolution kernels of size 3 × 3, which is followed by a 3 × 3 max-pooling layer and a batch normalization layer. Next, there is an Inception structure, which is followed by another max-pooling layer. The second module, namely, the “cascade dense Inception module,” is composed of four Inception structures with dense connections. The application of the dense connectivity strategy improves the usage efficiency of feature maps and promotes the fusion of multi-dimensional features among the Inception structures, enhancing the diagnostic performance for grape leaf diseases. The last module is composed of two max-pooling layers, an Inception layer, a global average pooling (GAP) layer, and a 7-way Softmax layer.

**Table 3:** Composition of the DICCN model.

Type	Patch size/stride	Output size
Deep separable convolution	3 × 3/1	224 × 224 × 64
Max-pooling	3 × 3/2	112 × 112 × 64
Deep separable convolution	3 × 3/1	112 × 112 × 64
Max-pooling	3 × 3/2	56 × 56 × 64
Batch normalization	–	56 × 56 × 64
Inception	–	56 × 56 × 576
Max-pooling	3 × 3/2	28 × 28 × 576
Inception	–	28 × 28 × 576
Inception	–	28 × 28 × 576
Inception	–	28 × 28 × 576
Inception	–	28 × 28 × 576
Max-pooling	3 × 3/2	14 × 14 × 576
Inception	–	14 × 14 × 960
Max-pooling	3 × 3/2	8 × 8 × 960
GAP	–	960
Softmax	–	7

### Deep Separable Convolutional Layer

Limited by the number of images of the grape leaf disease data set, the model with a large size is prone to overfitting during the training process. Therefore, reducing the number of parameters contributes to improve the generalization performance of the model. Furthermore, a model with fewer parameters has a higher training speed and consumes fewer computing resources. While, the deep separable convolution consists of a depthwise convolution and a pointwise convolution, which has fewer parameters than a standard convolution (Howard et al., 2017). In deep separable convolution, the single filter is applied in depthwise convolution to each input channel. Then, a 1 × 1 convolutional operation is applied by the pointwise convolution to combine the outputs. This factorization significantly reduces the model size and the consumption of computing resources, while the recognition accuracy of the model will not decrease.

### Cascade Dense Inception Module

**Figure 4:**
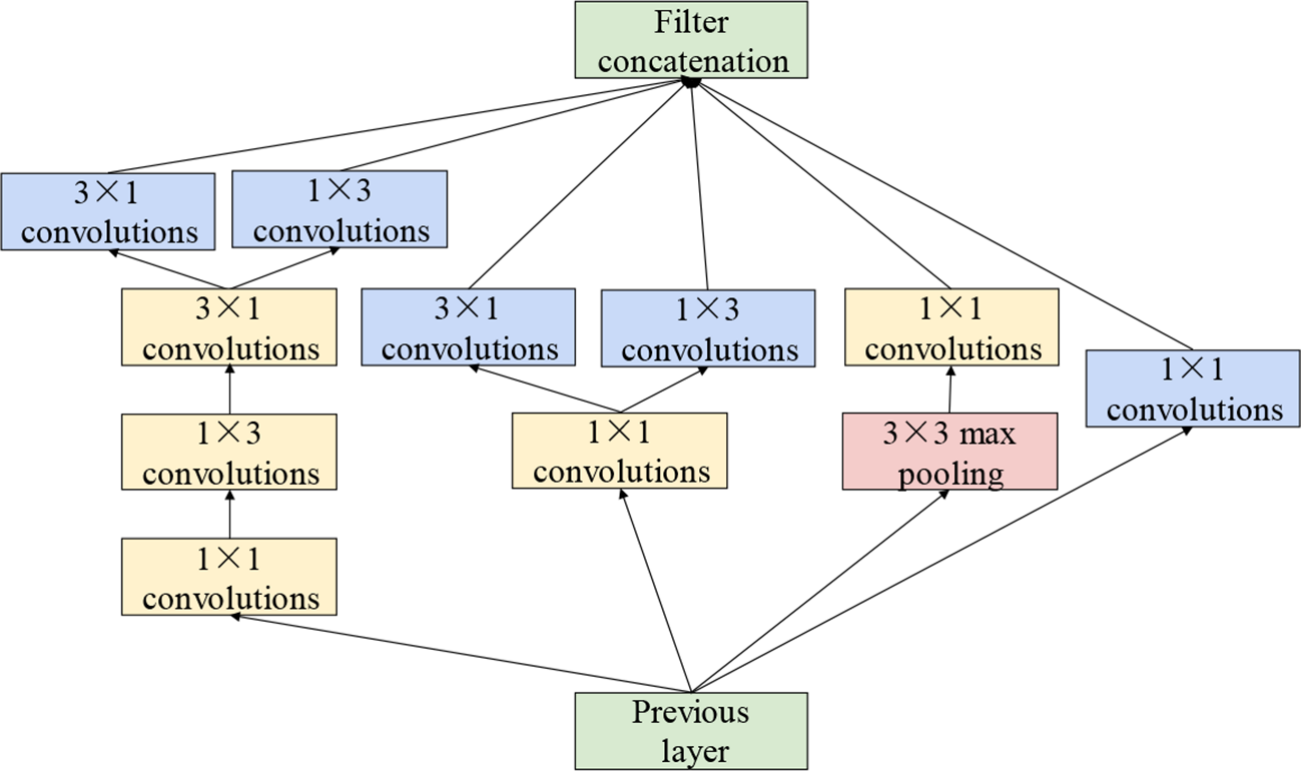
Inception structure.

The sizes of the disease spots differ substantially among types of grape leaves. The performance of the model in the extraction of features at various scales has a substantial impact on the final recognition accuracy. For extracting features of various sizes, the cascade dense Inception module, which is composed of four Inception structures with dense connections, has been applied to the model. The convolution kernels with small size extract fine-grained lesion features, whereas the convolution kernels of large size focus more on the features of disease spots of large size. Therefore, the Inception structure in GoogLeNet (Szegedy et al., 2015) has been applied. Inception structure stacks convolutional layers with different size on its branches in parallel. Each parallel branch of the Inception structure concentrates on distinct features. This not only increases the width of the network but also enhances the multi-scale feature extraction performance. In addition, based on asymmetric factorization approach (Szegedy et al., 2016), asymmetric convolutions are applied to strengthen the feature extraction performance and to reduce the computational cost. The Inception structure is illustrated in detail in **Figure 4**.

Generally, Floating-Point Operations is used to evaluate the time complexity of the CNN model. For a single convolutional layer, its time complexity can be expressed as:

$$Time∼O\left(M^{2}*K^{2}*C_{in}*C_{out}\right)$$

where M represents the side length of the output feature map, K represents the side length of the convolution kernel, C_in_ is the number of channels of the input feature map and C_out_ is the number of channels of the output feature map.

The Inception structure contains several convolutional layers and its time complexity can be expressed by the sum of operation time of all the convolutional layer:

$$Time∼O\left(\sum_{i=1}^{D}M_{i}^{2}*P_{i}*Q_{i}*C_{\left(i,in\right)}*C_{\left(i,out\right)}\right)$$

where D represents the number of convolutional layers in the Inception structure, P_i_ represents the length of the convolution kernel, Q_i_ represents the width of the convolution kernel (Q_i_ is not equal to P_i_ when asymmetric convolution is used).

During the flow of the feature maps, the features of small-scale grape disease spots are difficult to transfer to the deeper layers of the model. This loss of features severely affects the model’s recognition accuracy. In DenseNet, the dense connectivity strategy was proposed for further improving the information flow among layers. The *λ* layer obtains the feature maps from all preceding layers, as expressed in Equation:

$$x=H_{\lambda }\left(\left[\begin{array}{ccc} x_{0}, & x_{1}, & \begin{array}{cc} \ldots , & x_{\lambda -1} \end{array} \end{array}\right]\right)$$

where [*x*_0_,*x*_1_, …, *x_λ_*_-1_] denotes the concatenation of the maps from the previous layers.

**Figure 5:**
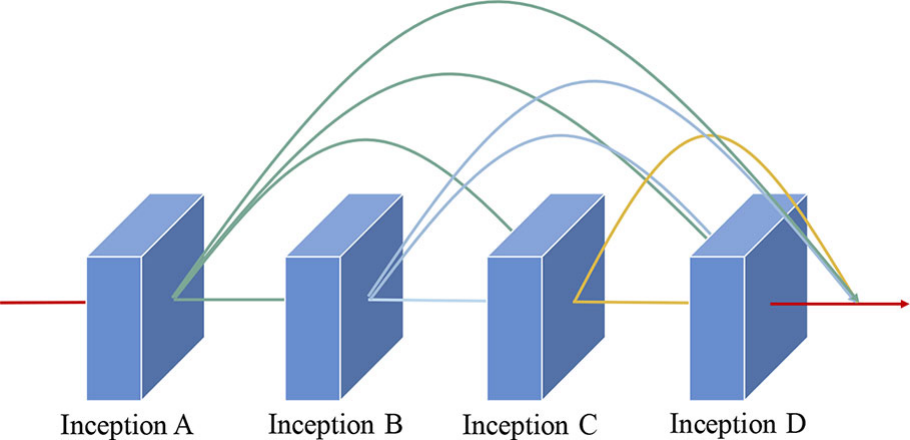
Schematic diagram of the dense connectivity strategy.

As illustrated in **Figure 5**, the dense connectivity strategy is applied to the cascade dense Inception module. Hence, the feature maps of all previous layers in this module are applied as inputs for this layer, and its own feature maps are applied as inputs for all subsequent layers. The application of the dense connection strategy is crucial for the improvement of model performance. First, the gradient that is obtained by each layer is the sum of the gradients from the previous layers; hence, it alleviates the vanishing-gradient problem. Furthermore, it strengthens feature propagation and encourages feature reuse, which can effectively prevent the overfitting problem. Finally, compared with the residual strategy, it substantially reduces parameters and the storage overhead of the proposed model.

### Adaptive Connectivity Strategy

A CNN-based model must be trained for the classification of grape leaf diseases. The choice of the optimization algorithm has a substantial influence on the training performance.

Adaptive moment estimation (Adam) was applied instead of Stochastic gradient descent (SGD), a traditional algorithm, as the optimization algorithm of the model. Adam is an efficient algorithm for the first-order gradient-based optimization of stochastic objective functions (Kingma and Ba, 2015). The algorithm has low memory requirements, and it is simple to implement; hence, it is suitable for problems with large amounts of data or many parameters. The updated weights are calculated based on the previous iteration, and the process of weight optimization is expressed as:

$$g_{t}=\nabla_{\theta }f_{t}\left(\theta_{t-1}\right)$$

$$m_{t}=\beta_{1}\cdot m_{t-1}+\left(1-\beta_{1}\right)\cdot g_{t}$$

$$v_{t}=\beta_{2}\cdot v_{t-1}+\left(1-\beta_{2}\right)\cdot g_{t}^{2}$$

$${\hat{m}}_{t}=m_{t}/\left(1-\beta_{1}^{t}\right)$$

$${\hat{v}}_{t}=v_{t}/\left(1-\beta_{2}^{t}\right)$$

$$\theta_{t}=\theta_{t-1}-\alpha \cdot {\hat{m}}_{t}/\left(\sqrt{{\hat{v}}_{t}}+\varepsilon \right)$$

where *α* represents the learning rate, *β*_1_ and *β*_2_ represent the exponential decay rates for the moment estimates, *θ*_t_ is the current updated parameter, *θ*_t-1_ is the previous updated parameter, *f*(*θ*) represents a stochastic function with parameters *θ*, *ε* is a small constant (*ε*=10^-8^ in this paper), m_t_ is the first moment vector, and *v*_t_ is the second moment vector.

## Experimental Results and Discussion

### Adaptive Connectivity Strategy

The experiments were conducted on a deep learning server that contained two Tesla P100 processors (16 GB memory) with an Ubuntu system. In addition, the TensorFlow and Keras deep learning frameworks were used to implement the DICNN model, which is convenient for the development of comparative experiments due to its Python interfaces (Bahrampour et al., 2015; Abadi et al., 2016a; Abadi et al., 2016b; Tang, 2016). Additional configuration parameters are listed in **Table 4**.

**Table 4:** Software and hardware environment.

Configuration	Value
Central processing unit	Intel^®^ Xeon CPU E5-2650 v4 @ 20 GHz ^×^ 48
Graphics processor unit	NVIDIA Tesla P100-PCIE-16 GB ^×^ 2
Operation system	Ubuntu 16.04.2 LTS (64-bit)
Deep learning framework	TensorFlow, Keras

### Accuracy and Convergence Speed Comparisons

Based on the test set, an experiment is conducted to compare the accuracy and convergence speed of the DICNN model with other classical approaches, including the back-propagation (BP) neural network, support-vector machine (SVM), VGG-16, GoogLeNet, ResNet-34, and DenseNet-169. Meanwhile, the proposed model is also compared with the recent model on grape diseases classification, including AlexNet for grape diseases classification (AFGDC) (Wagh et al., 2019) and UnitedModel (Ji et al., 2019).

**Figure 6:**
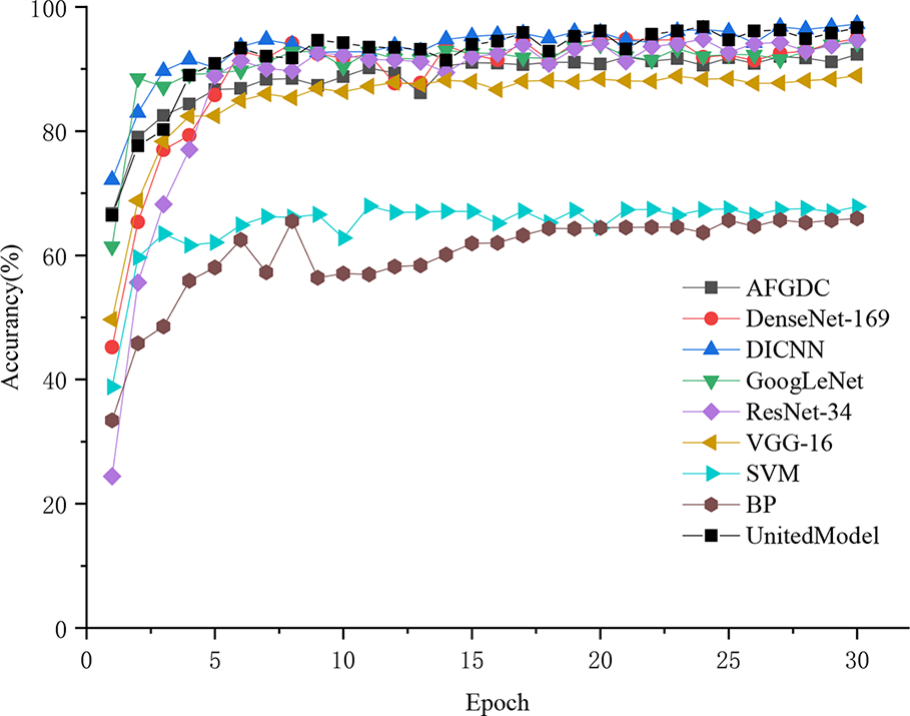
Convergence of eight recognition models.

All classification models were trained from scratch with 30 epochs, and the same training strategy was adopted. The Adam algorithm was used as the optimizer for the model training. And the learning rate was set to 0.01, which can accelerate the convergence of the model during the training process. According to **Table 5**, the proposed DICNN model had optimal recognition performance with an accuracy of 97.22% on the test set. In addition, an accuracy of 94.89% was realized by DenseNet, which is due to its compelling advantages of strengthening feature propagation and encouraging feature reuse. ResNet-34, a residual neural network, realized an overall accuracy of 94.89%. In addition, GoogLeNet realized an accuracy of 94.25%, which is due to its multi-dimensional feature extraction capabilities. VGG-16 obtained an average accuracy of 88.96%, whereas the SVM model and BP neural network exhibited poor recognition performances, with the accuracy of 67.82% and 57.93%, respectively. UnitedModel, which was specially designed for grape disease detection, was able to extract complementary discriminative features owing to the combination of multiple CNNs. And it realized an accuracy of 96.58%. Another grape disease detection model, AFGDC, used pre-defined AlexNet architecture for feature extraction and achieved 88% accuracy. The experimental results demonstrated that the CNN-based approaches outperform the classical machine learning approaches. The classical machine learning approaches in grape leaf disease recognition depend on classification features, which are designed by experts. In contrast, the CNN-based approaches extract the best classification features automatically. With those features, CNN-based models realize excellent recognition performance on grape leaf diseases. Among all CNN models, DICNN have better performance and can accurately classify grape disease images. In addition, accuracy curves were used to visually represent the accuracies and convergence speeds of the models. As shown in **Figure 6**, the models have converged after several epochs rounds of training and ultimately realized their optimal identification performances. Overall, the training processes of DenseNet-16, DICNN, GoogLeNet, ResNet-34, UnitedModel, AFGDC, and VGG-16 are approximately stable after 9 epochs, and the BP neural network and the SVM model showed acceptable convergence after 17 epochs. In our work, the dense connectivity strategy and Inception structures were adopted for the proposed DICNN model. Compared with other models, the proposed DICNN model realized the fastest convergence rate and tended to converge at the sixth epoch.

**Table 5:** Recognition Performance.

Model	BP	SVM	VGG-16	GoogLeNet	ResNet-34	DenseNet-169	UnitedModel	AFGDC	DICNN
Accuracy	65.93%	67.82%	88.96%	94.25%	94.67%	94.89%	96.58%	92.33%	97.22%

Furthermore, unseen images with different grape farms and weather conditions are collected and used to test the generalization performance of the model. Those grape leaf disease images are collected from the grape planting base of Yuanshi Chateau, which is Yinchuan City, Ningxia Province, China. According to the final experimental results, the DICNN model has 96.86% accuracy when tested with unseen images. Although the accuracy of the model is slightly lower than before, the model can still accurately classify grape leaf diseases. Therefore, the experimental results have proved that the model have excellent generalization performance in different grape farms and weather conditions.

With its Inception structure, DICNN can extract features from multiple scales based on the characteristics of grape leaf lesions. Using deep separable convolution effectively reduces the parameters of CNN model, thus alleviates the problem of overfitting. In addition, with the dense connectivity strategy of DICNN, feature propagation is enhanced, and feature reuse is encouraged. Hence, the proposed algorithm gives better performance than popular transfer learning techniques.

### Recognition Performance for Each Class

In this section, based on the confusion matrix, the recognition performance of each grape leaf disease has been evaluated by Precision, Recall and F1 Score. Confusion matrix, as a standard format for expressing accuracy evaluation, is expressed by matrix form with n rows and n columns. Each column of the confusion matrix stands for the number of instances in a ground truth class while each row stands for the number of instances in a predicted class to see if the system is confusing two classes. Precision, Recall and F1 Score are derived from the number of false positive (FP), true positive (TP), false negative (FN), and true negative (TN) results. These indicators are derived as follows:

$$Precision=\frac{TP}{TP+FP}$$

$$Recall=\frac{TP}{TP+FN}$$

$$F1\;Score=\frac{2\times Precision\times Recall}{Precision+Recall}=\frac{2\times TP}{2\times TP+FN+FP}$$

**Table 6** presents the confusion matrix of the final test results and the Precision, Recall and F1 Score of each type of grape leaves. The disease spots of leaf diseases are similar in terms of geometrical features, leading to lower classification performance. Hence, the classifier may misjudge when faced with fine-grained classification. However, the proposed deep learning model has yielded a satisfactory result. The main feature of the mites class is that the surfaces of the leaves are blistered, which differs significantly from the spots of the other diseases. Confirmed from the confusion matrix, the diagnosis of mites is better than others. The color of the downy mildew spots is yellow-green, so these spots are easy to distinguish from those of other diseases. Hence, the Recall of downy mildew reaches 98.04%. However, brown spots, anthracnose, leaf blight and black rot are similar in terms of their geometric features, and this similarity leads to their lower recognition rates. The Recall of the brown spot, anthracnose, leaf blight and black rot classes were 96.54%, 95.84%, 97.05% and 97.29%, respectively. Ultimately, 96.60% of healthy leaves were correctly identified.

**Table 6:** Confusion matrix of the DICNN model.

	Class	Predicted							Precision	Recall	F1 Score
		Anthracnose	Brown spot	Mites	Black rot	Downy mildew	Leaf blight	Healthy leaves			
Ground Truth	Anthracnose	3,016	32	15	34	10	26	14	96.57%	95.84%	0.9620
	Brown spot	29	3,738	5	35	26	28	11	97.32%	96.54%	0.9693
	Mites	3	2	3,078	3	5	2	4	98.81%	99.39%	0.9910
	Black rot	28	28	4	3,234	4	16	10	96.28%	97.29%	0.9719
	Downy mildew	8	11	3	17	2,498	7	4	96.90%	98.04%	0.9762
	Leaf blight	23	18	6	22	9	2,924	11	96.98%	97.05%	0.9739
	Healthy leaves	16	12	4	14	26	12	2,388	97.79%	96.60%	0.9751

Based on the Inception structure, the disease features in the original image can be extracted from multiple dimensions. Thus, the accuracy of disease image recognition is significantly increased. Supported by the above experiments, the proposed DICNN model realizes superior recognition performance in identifying grape leaf diseases.

### Effect of Data Augmentation on Identification Performance

In this paper, data augmentation has been utilized to prevent overfitting. First, the diseased grape leaves were captured under various weather conditions. By changing the shooting background, the anti-interference performance against complex conditions of the proposed model can be enhanced. Afterward, digital image processing techniques were used to augment the original data set.

**Figure 7:**
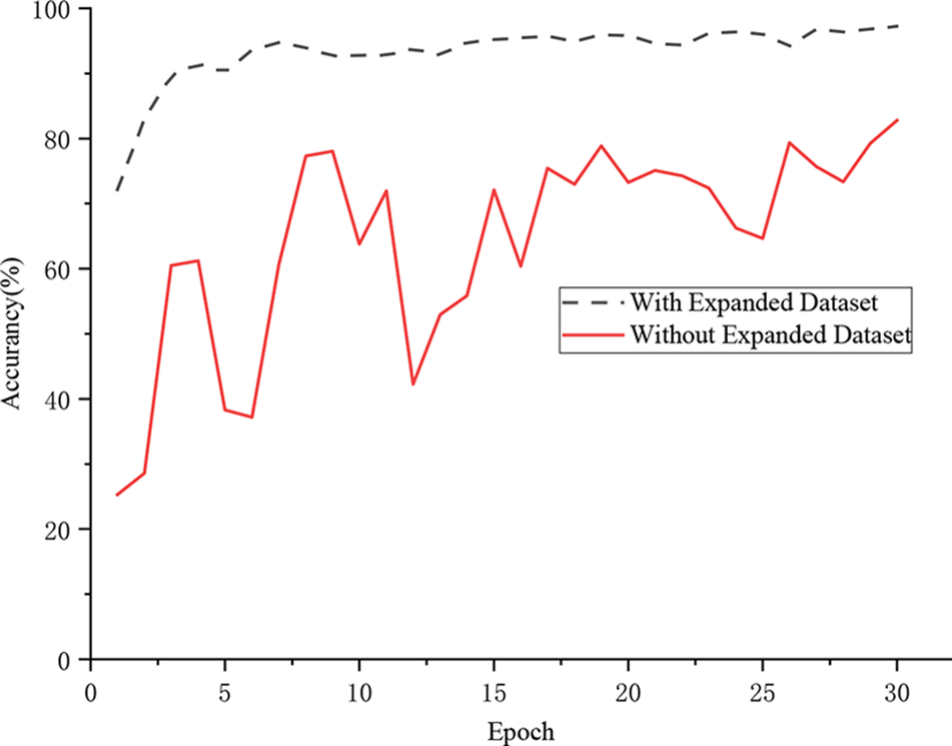
Effect of data augmentation.

In this section, a comparative experiment was designed for evaluating the influence of data augmentation on the classification accuracy. **Figure 7** shows that the proposed DICNN model had an extremely unstable training process when training on the original data set. The model finally realized a recognition rate of 82.80%. However, the model that was trained on the expanded data set realized an accuracy of 97.22%. The experimental results demonstrated that the DICNN model learns more suitable features on the expanded data set, which enhances the anti-interference performance under various environments. In addition, the parameters of the classification model were fully trained due to the diversity of images in the extended data set, while the images in the original data set were lacking in diversity, which made the network model overly dependent on a subset of the features, thereby resulting in overfitting. More importantly, the pre-processing of the image simulated the real environment of the grape leaves, thereby making the model more robust.

### Effect of Dense Connectivity Strategy

**Figure 8:**
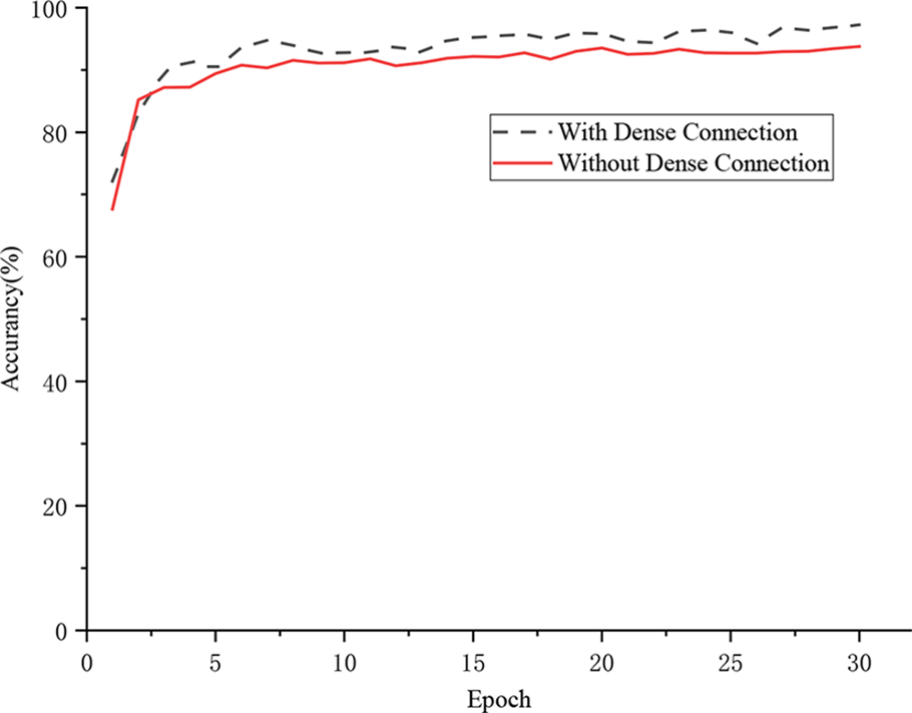
Effect of the dense connectivity strategy.

This experiment evaluated the influence of the dense connection strategy on the recognition performance of the CNN-based model. As shown in **Figure 8**, under the same training strategy, the model with the dense connection strategy realized 97.22% recognition accuracy, which was 3.47% higher than that of the model in which the dense connection strategy was not applied. The dense connection strategy connects Inception structures in the convolutional layer to ensure maximum information transmission among Inception structures in the network and directly transfers the gradient loss to the shallow layers. Therefore, the proposed model with this strategy realizes stronger performance in the identification of grape leaf diseases.

### Effect of Deep Separable Convolutional Layer

Deep separable convolution is used by DICNN to build the first two convolutional layers to reduce parameters and prevent the overfitting problem of the model. In order to evaluated the influence of two deep separable convolutional layer, the model with traditional convolutional layer has been trained. In the comparative experiment, the number of parameters of the convolutional layers and the recognition accuracy are used as the final evaluation indicators. The final experimental results are shown in **Table 7**. On one hand, the parameters of the first convolutional layer was reduced from 1,792 to 283, and the parameters of the second convolutional layer was reduced from 36,928 to 4,736, which contributes to reduce the consumption of computing resources and improve the generalization performance. On the other hand, the accuracy of the model is improved by 0.13% compared with models containing traditional convolutional layers.

**Table 7:** Effect of deep separable convolutional layer.

	# Parameters			Accuracy
	1st convolution layer		2nd convolution layer	
Model with deep separable convolutional layer	283	4,736		97.22%
Model with traditional convolutional layer	1,792	36,928		97.09%

### Optimization Selection

The choice of the optimization algorithm is crucial for the improvement of model performance. Therefore, the Adam optimization algorithm was adopted for the proposed DICNN model. The Adam optimization algorithm and the SGD optimization algorithm with the same learning rate of 0.01 were applied to train the DICNN model for evaluating the performance of the algorithm.

**Figure 9:**
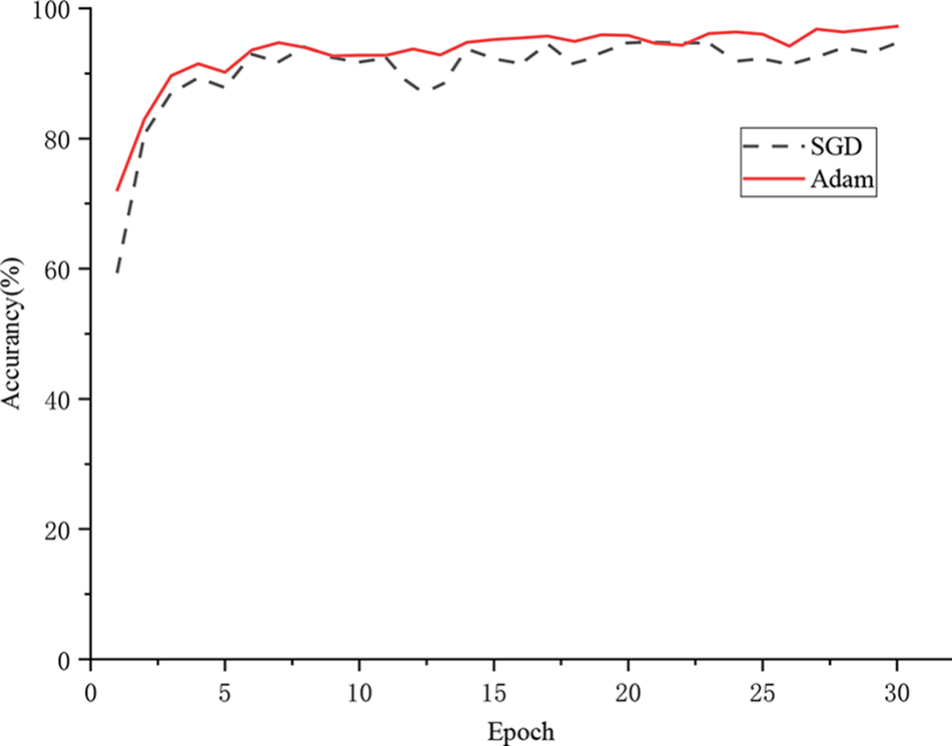
Comparison of two optimization algorithms.

**Figure 9** shows the training process of the model. The accuracy of the model with the Adam optimization algorithm is 97.22%, while the accuracy of the model with the SGD optimization algorithm is 94.69%. The SGD optimization algorithm updated the parameters based on the current position and batch, which led to an extremely unstable direction of updating. According to the experimental results, the model that is based on the SGD optimization algorithm encountered a “local minimum” problem and was unable to reach the optimal state. The Adam optimizer utilizes gradient descent with momentum to escape from the local minimum position. In addition, the Adam optimization algorithm is an adaptive optimization scheme, which adjusts the learning rate for each parameter. Therefore, the Adam optimization algorithm was adopted for the proposed DICNN model.

### Feature Visualization Process

Due to the weak interpretive performance, the features that are learned by CNN-based models are difficult to represent in a human-readable form. Hence, it is challenging to comprehend the massive number of parameters, the multi-layer hidden structure, and other factors of these models. However, visualization techniques are a prominent way to explore how CNNs learn features for distinguishing among classes. In this section, the two most commonly used visualization techniques, namely, visualization of intermediate activation and visualization of heatmaps of class activation in an image, are used to analyze the proposed model.

**Figure 10:**
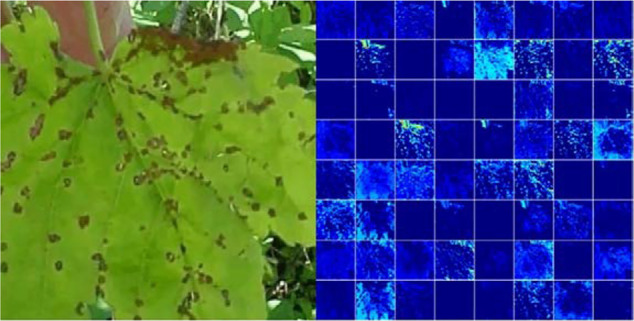
Activation visualization.

The visualization of intermediate activation refers to the display of feature maps which are output by all kinds of convolution and pooling layers in the network for a specified input. This facilitates understanding of how successive convolution layers transform their input and of the meaning of each filter. **Figure 10** shows an original image from the anthracnose class and the visual activation image after the second convolutional layer of the DICNN model. According to the visualization results, the disease spot area of the grape leaf is clearly separated from the background in the image. It is inferred that the model can identify disease spots in the image and can characterize the disease spots as one of the criteria for classification. The experiment of the activation visualization for grape leaf diseases illustrates the superior recognition performance of the DICNN model and show how the proposed DICNN model learns features for distinguishing between the lesion area and the background.

**Figure 11:**
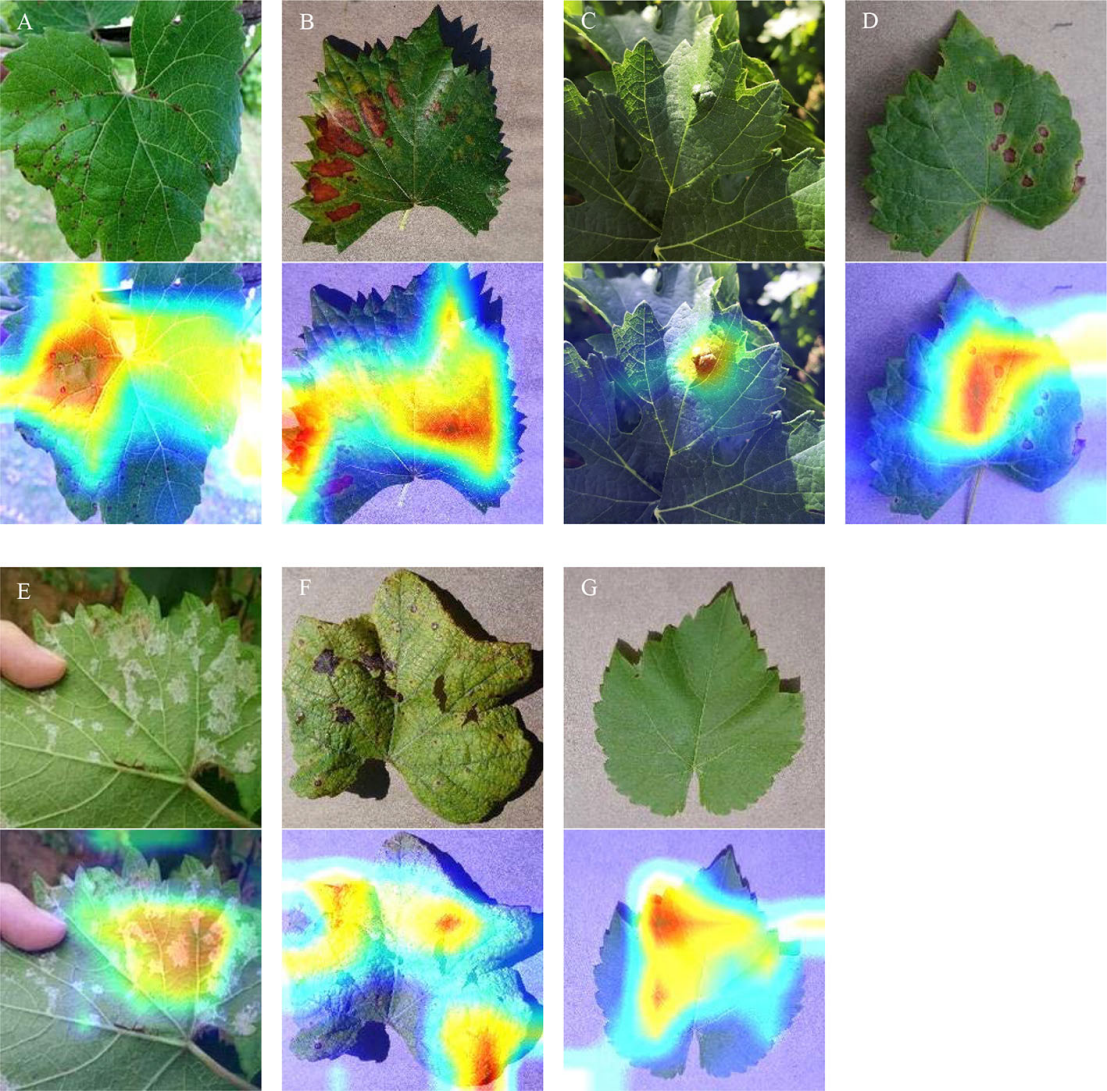
CAM visualization. **(A)** Anthracnose; **(B)** Brown spot; **(C)** Mites; **(D)** Black rot; **(E)** Downy mildew; **(F)** Leaf blight; **(G)** Healthy leaves.

The visualization of heatmaps of class activation refers to the production of heatmaps of class activation over input images (Selvaraju et al., 2017). These visualization techniques are also known as class activation map (CAM) visualization techniques, which facilitate understanding of which parts of the input image lead to the final classification decision of the convolutional neural network. **Figure 11** shows an original image and the generated heatmaps of class activation. Those visualized data facilitate understanding of which parts of the input image lead to the final classification decision of the model. According to the visualization results, the disease spot area is strongly activated: this is how the network distinguishes different grape leaf diseases. Overall, the results of this experiment demonstrate that the model pays full attention to the features of the disease spot and realizes superb recognition performance on grape leaf diseases.

## Conclusions

This paper has proposed a deep learning approach for the identification of six common grape leaf diseases and healthy leaves. Based on 7,669 collected grape leaf images, 107,366 images were created *via* image augmentation. By analyzing the features of grape leaf diseases, an improved CNN is proposed for the identification of grape leaf diseases. The deep separable convolution was applied to the model instead of the standard convolution to alleviate overfitting and reduce the number of parameters. In view of the various sizes of grape leaf disease spots, Inception structures were applied to the model for enhancing ability of the multi-scale feature extraction. In addition, the dense connectivity strategy was introduced for encouraging feature reuse, strengthening feature propagation.

The proposed CNN-based identification approach for grape leaf diseases was implemented in the TensorFlow and Keras frameworks on the Tesla P100 GPU platform. With the expanded data set, the proposed DICNN model was trained to classify seven type grape leaves. According to the experimental results, the proposed algorithm realizes a recognition accuracy of 97.22%, which gives better performance than other popular transfer learning techniques. Compared with the standard ResNet and GoogLeNet architectures, the proposed DICNN model realizes higher convergence speed during the training process and higher accuracy. The results of this study demonstrate that the proposed algorithm realizes end-to-end classification of grape leaf diseases and provides a solution and a reference for the application of deep learning approaches in the classification of crop diseases.

## Data Availability Statement

The datasets generated for this study are available on request to the corresponding author.

## Author Contributions

Conceptualization, BL, ZD, and LT. Methodology, BL, ZD, and LT. Software, ZD and LT. Validation, BL, DH, SL, and HW. Writing—original draft preparation, ZD and LT. Writing—review and editing, BL, ZD, and LT. Supervision, BL. Funding acquisition, BL, DH, SL, and HW. All authors contributed to the article and approved the submitted version.

## Funding

This research is supported by the National Natural Science Foundation of China under Grant No. 61602388, by the China Postdoctoral Science Foundation under Grant No. 2017M613216, by the Fundamental Research Funds for the Central Universities under Grant No. 2452019064, by the Natural Science Basic Research Plan in Shaanxi Province of China under Grant No. 2017JM6059, by the Postdoctoral Science Foundation of Shaanxi Province of China under Grant No. 2016BSHEDZZ121, by the Ningxia Smart agricultural Industry Technology Collaborative Innovation Center under Grant No. 2017DC53, by the Key Research and Development Program of Shaanxi under Grant No. 2019ZDLNY07-06-01, and by the Innovation and Entrepreneurship Training Program of Northwest A&F University of China under Grant No. 201910712048.

## Conflict of Interest

Author HW was employed by the company West Electronic Business.

The remaining authors declare that the research was conducted in the absence of any commercial or financial relationships that could be construed as a potential conflict of interest.
